# Neglected intrapulmonary arteriovenous anastomoses: A comparative study of pulmonary right-to-left shunts in patients with patent foramen ovale

**DOI:** 10.3389/fcvm.2023.1111818

**Published:** 2023-04-06

**Authors:** Anni Chen, Jianbo Zhu, Lei Zhu, Yunyi Tang, Yun Li, Qi Zhang, Yeping Zhao, Caiye Ma, Xiatian Liu

**Affiliations:** ^1^ Department of Ultrasound, The First Affiliated Hospital of Shaoxing University, Shaoxing, China; ^2^ Department of Ultrasound, Shaoxing People’s Hospital, Shaoxing, China

**Keywords:** pulmonary right-to-left shunt, patent foramen ovale, intrapulmonary arteriovenous anastomoses, transthoracic echocardiography, transesophageal echocardiography, saline contrast echocardiography

## Abstract

**Objective:**

Pulmonary right-to-left shunt (P-RLS) and patent foramen ovale right-to-left shunt (PFO-RLS) often appear in combination, and there are often differences and connections between them. Intrapulmonary arteriovenous anastomoses (IPAVAs), as part of P-RLS, are often overlooked because there are no technologies to detect and identify them. This study aimed to further clarify the incidence and characteristics of P-RLS with the help of contrast transesophageal echocardiography (c-TEE) and contrast transthoracic echocardiography (c-TTE), providing a reference for clinically relevant research and patent foramen ovale (PFO) management disposal decisions.

**Methods:**

We retrospectively investigated 414 subjects who came to our hospital for c-TEE from October 2021 to July 2022, and all subjects completed c-TTE simultaneously. 7 Patients who were newly diagnosed with an atrial septal defect were excluded. Eventually, 407 patients were included in this study. Among them, 157 patients with PFO (58 patients were treated with PFO closure subsequently) and 250 patients without PFO confirmed by c-TEE were finally enrolled. In the process, we observed and analysed the presence of P-RLS.

**Results:**

A total of 407 patients were included in the final analysis and divided into PFO group (*N* = 157) and non-PFO group (*N* = 250) according to the results of c-TEE. Whether at rest or after Valsalva maneuver, the incidence of P-RLS was significantly higher under c-TEE than under c-TTE in the two groups (*P* < 0.001). For both c-TTE and c-TEE, the incidence of P-RLS was slightly higher after Valsalva maneuver than at rest, but the difference was not significant (c-TTE: rest vs. Valsalva maneuver, *P* = 0.214; c-TEE: rest vs. Valsalva maneuver, *P* = 0.076). The Valsalva maneuver increased the incidence of P-RLS in the group without PFO, which was more significant in c-TEE (c-TTE: rest vs. Valsalva maneuver, *P* = 0.591; c-TEE: rest vs. Valsalva maneuver, *P* = 0.008). In both groups, the P-RLS semiquantitative grading was statistical significance under different states and examinations (*P* < 0.001).

**Conclusion:**

The vast majority of P-RLS are grade 1–2 and are derived from physiological IPAVAs. Even so, attention should be given to the differentiation between P-RLS and PFO-RLS. c-TEE is an effective method to detect P-RLS; however, the recruitments of c-TEE and Valsalva maneuver to P-RLS should be noted.

## 1. Introduction

Patent foramen ovale (PFO) is defined as the failure of the septum primum to completely fuse with the septum secundum within 3 years of birth and when there is a potential lacuna formed at the fossa ovalis of the interatrial septum (1). In some cases, such as Valsalva maneuver, coughing, sneezing, laughing, defecation, and weight-bearing increase the thoracic pressure, followed by increases in the right atrial pressure, which causes a higher pressure in the right atrium than the left atrium. Then, the fossa ovalis channel opens or the original gap expands further, which can lead to varying degrees of right-to-left shunt (RLS) (2). RLS is divided into intracardiac shunt and extracardiac shunt, the former mainly refers to patent foramen ovale right-to-left shunt (PFO-RLS), while the latter is pulmonary right-to-left shunt (P-RLS), including physiological intrapulmonary arteriovenous anastomoses (IPAVAs) and rare pulmonary arteriovenous malformations/fistulas (PAVMs). A large number of studies have found that RLS is associated with arterial embolic ischemic events, commonly stroke, migraine, transient ischemic attack, etc., and paradoxical embolism caused by RLS is the mechanism of its occurrence, it simply refers to venous and right heart emboli through the RLS into the systemic circulation, and severe cases can lead to disability and death (3, 4).

PFO-RLS has been considered one of the essential causes of cryptogenic stroke. PFO occurs in approximately 25% of normal adults (5). The incidence of PFO in patients with cryptogenic stroke is as high as 40% (6). Therefore, Elgendy proposed the concept of PFO-associated stroke in 2020 and recommended it as an independent classification of cryptogenic stroke (7). IPAVAs are micro arteriovenous channels present in the lungs of healthy individuals (8). Due to the lack of understanding of IPAVAs, and the diagnosis grey zone between IPAVAs with some PAVMs, P-RLS was mostly attributed to PAVMs. However, the incidence of PAVMs in the population is 2–3/100,000 (9), therefore, the study of P-RLS has been neglected. In recent years, some studies showed that P-RLS is a potential facilitator of stroke; at the same time, there is indeed a problem that P-RLS is regarded as PFO-RLS leading to overtreatment of PFO, they all remind us that P-RLS should not be ignored, IPAVAs in particular (10–12). In this study, we used contrast transesophageal echocardiography (c-TEE) and contrast transthoracic echocardiography (c-TTE) to compare P-RLS and PFO-RLS in order to investigate the characteristics of P-RLS and the relationship between P-RLS and PFO-RLS, hoping to provide new ideas and references for clinical follow-up studies and management disposal decisions of PFO.

## 2. Materials and methods

### 2.1. Patient population

**Figure 1:**
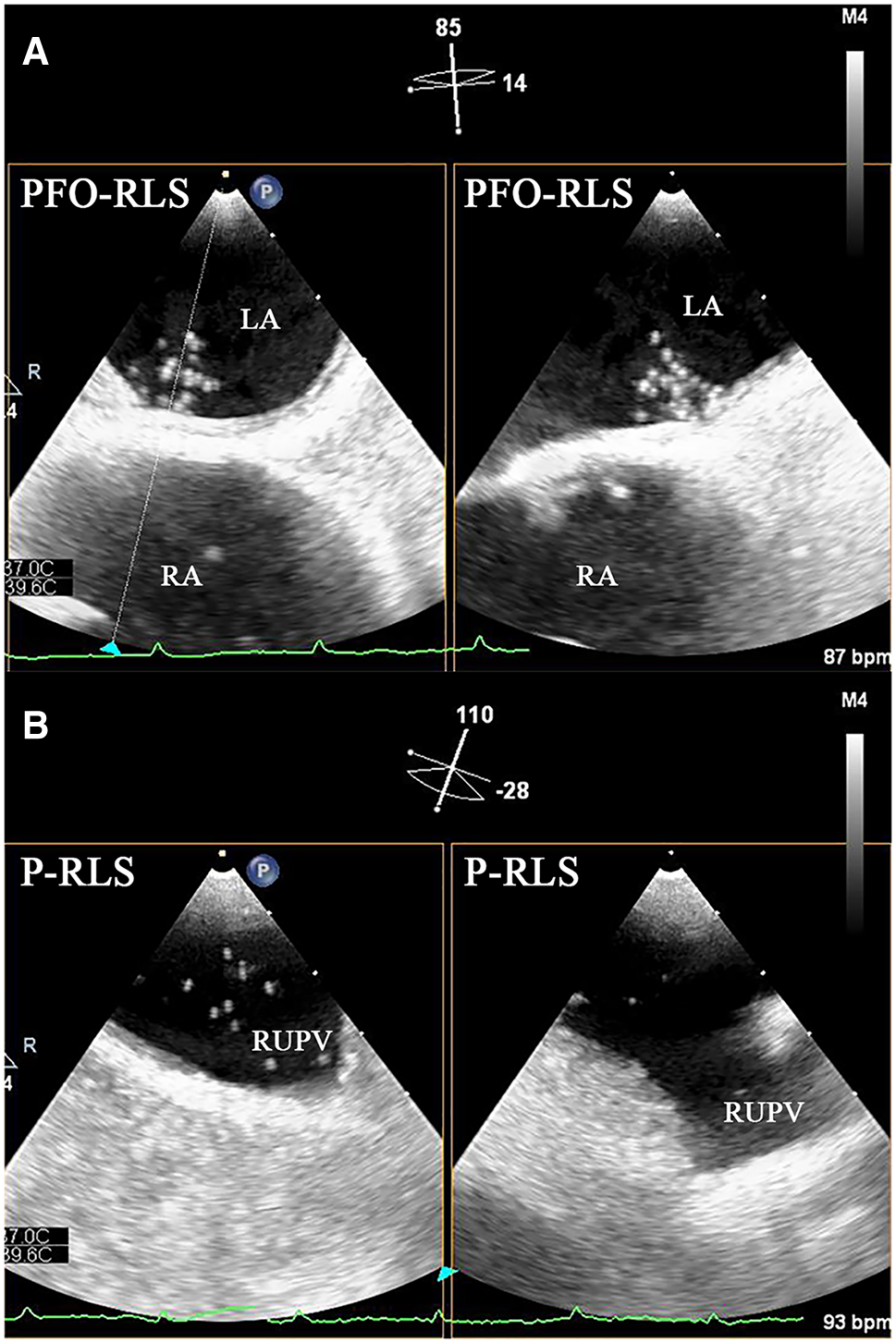
Biplane images of PFO-RLS and P-RLS under c-TEE. (**A**) PFO-RLS: microbubbles were observed to squeeze through the PFO. (**B**) P-RLS: microbubbles were observed to originate from the right superior pulmonary veins. PFO-RLS, patent foramen ovale right-to-left shunt; P-RLS, pulmonary right-to-left shunt; c-TEE, contrast transesophageal echocardiography; LA, left atrium; RA, right atrium; RUPV, right upper pulmonary vein.

**Figure 2:**
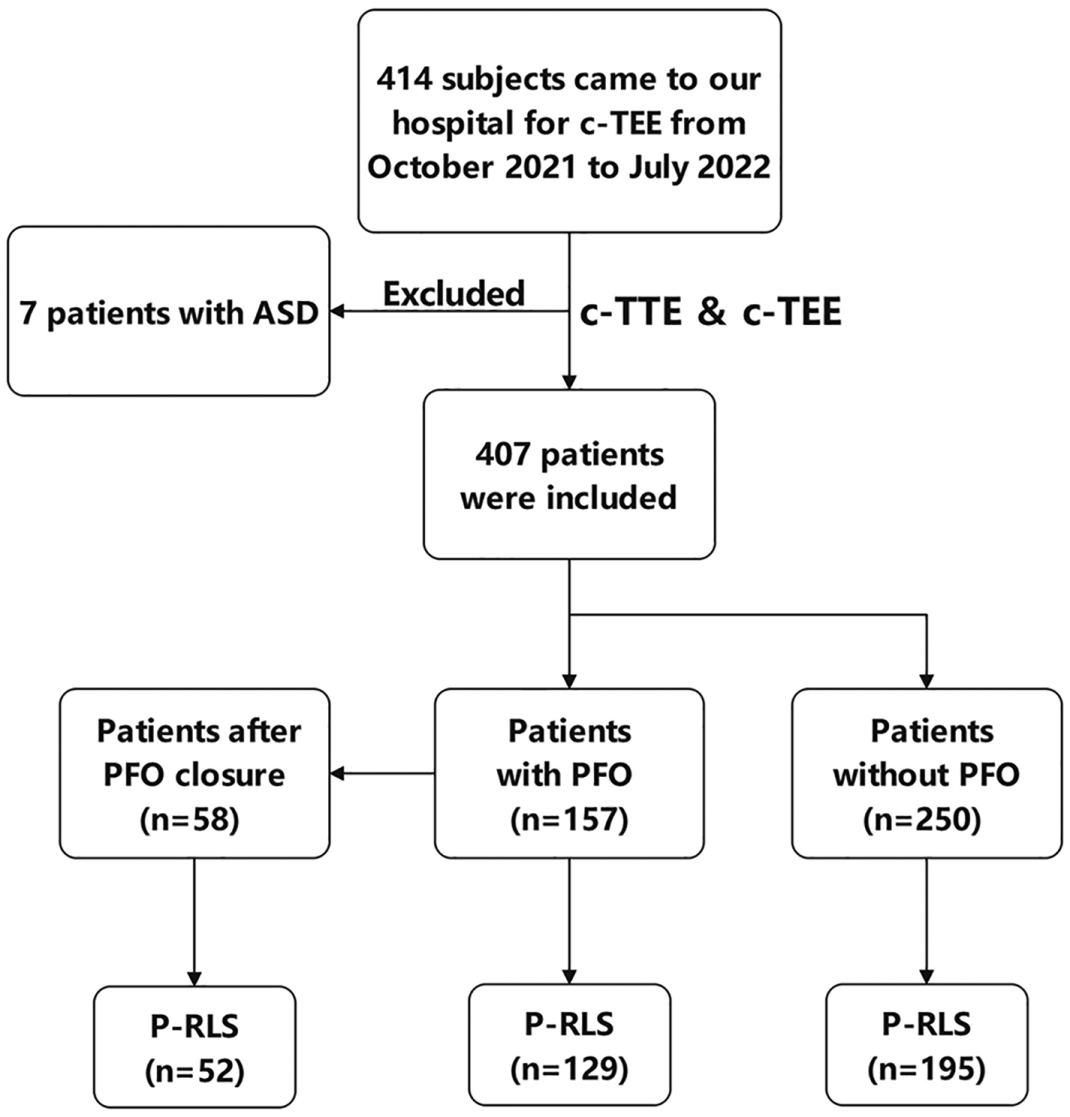
Flow chart of patient enrollment. c-TTE, contrast transthoracic echocardiography; c-TEE, contrast transesophageal echocardiography; ASD, atrial septal defect; PFO, patent foramen ovale; PFO-RLS, patent foramen ovale right-to-left shunt; P-RLS, pulmonary right-to-left shunt of patients after PFO closure; P-RLS, pulmonary right-to-left under c-TEE and after Valsalva maneuver.

We retrospectively investigated 414 subjects who came to our hospital for c-TEE from October 2021 to July 2022, and all subjects completed c-TTE simultaneously. In the process, we observed and analysed the presence of P-RLS and PFO-RLS. Figure 1 depicts the images of PFO-RLS and P-RLS under c-TEE. The criteria for diagnosing PFO is direct observation of microbubbles squeezing through the PFO under c-TEE. 7 Patients who were newly diagnosed with an atrial septal defect were excluded. The following patients were excluded: (1) those with contraindications to transesophageal echocardiography or saline contrast echocardiography, (2) those with cardiac organic diseases, such as congenital heart disease, atrial fibrillation, valvular disease, etc., (3) those with pulmonary hypertension or all diseases that can cause pulmonary hypertension, (4) those with cirrhosis to avoid the possibility of hepatopulmonary syndrome, and (5) those who could not cooperate with the examination. Written informed consent was obtained from each participant. Cryptogenic stroke was defined by the TOAST (Trial of Org 10172 in Acute Stroke Treatment) criteria and combined with cranial magnetic resonance imaging and neurological examination for diagnosis. Eventually, 407 patients were included in this study. Among them, 157 patients with PFO and 250 patients without PFO confirmed by c-TEE were finally enrolled (Figure 2). Subsequently, 58 PFO patients were treated with PFO closure, and all patients came to our hospital for c-TTE and c-TEE within 1 month before surgery and 3 months after surgery (13). The study was approved by the local ethics committee.

### 2.2. Equipment and operation methods

**Figure 3:**
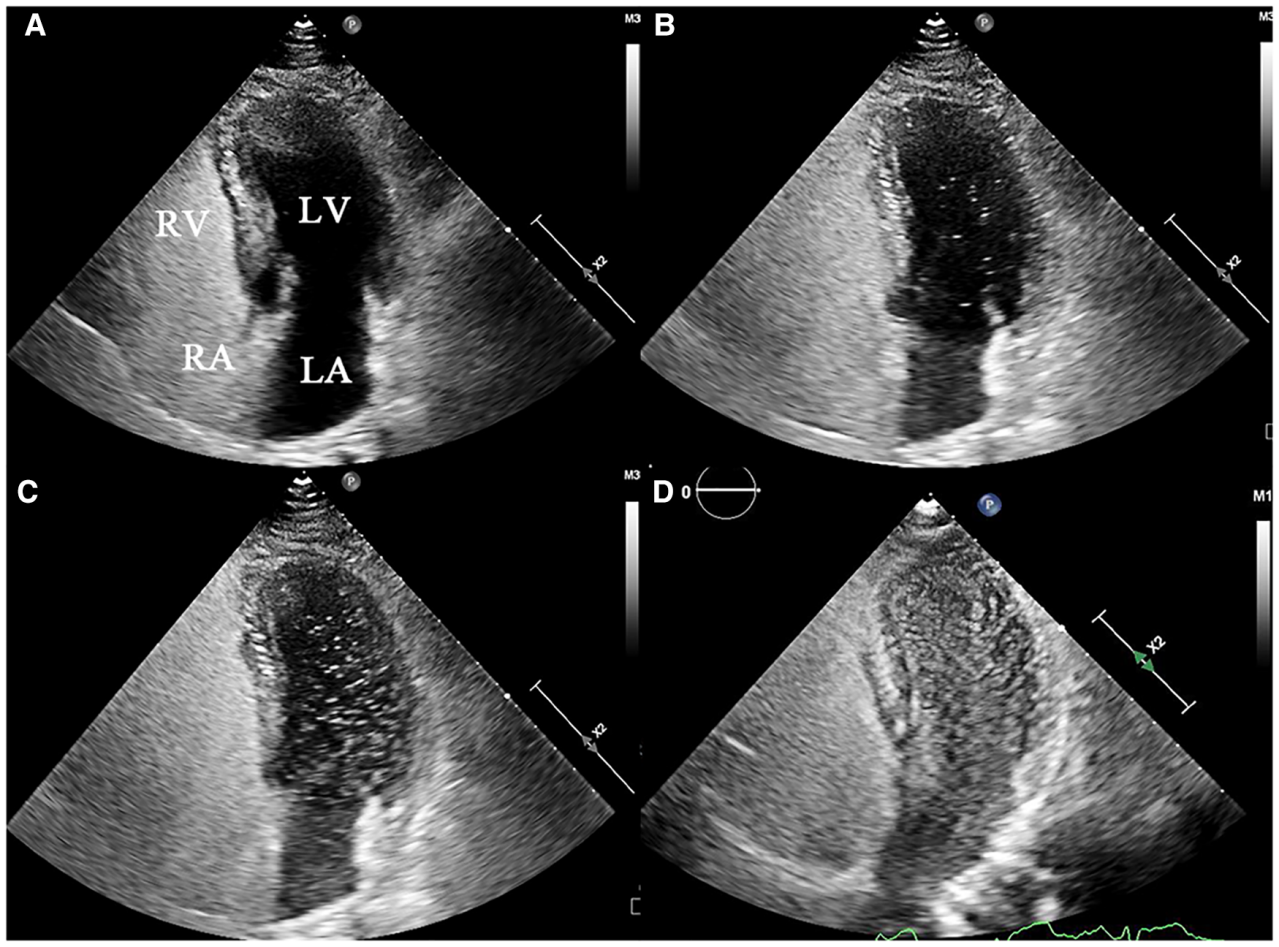
Representative echocardiograms of P-RLS grading under c-TTE (grade 0–3). (**A**) grade 0: negative in LV, (**B**) grade 1:1–30 bubbles in LV, (**C**) grade 2:31–100 bubbles in LV, (**D**) and grade 3:>100 bubbles in LV. P-RLS, pulmonary right-to-left shunt; c-TTE, contrast transthoracic echocardiography; LA, left atrium; RA, right atrium; LV, left ventricle; RV, right ventricle.

All c-TTE/c-TEE examinations were performed according to the local clinical protocols. A Philips EPIQ 7C real-time three-dimensional color cardiac ultrasound system (Philips Ultrasound, Bothell, Washington, United States) was used with a transesophageal three-dimensional matrix probe X8-2t (frequency 2–8 MHz) and a transthoracic three-dimensional pure wave matrix probe X5-1 (frequency 1–5 MHz). The contrast agent was an agitated saline solution. Two 10 ml syringes were utilized, one containing mixed saline (8 ml 0.9% sterile saline+1 ml air+1 ml venous blood of the subject), and the other was an empty tube connected to the former through a three-way tube. The two syringes were injected back and forth more than 10 times to fully mix and obtain saline-containing microbubbles. The agitated saline was injected through the antecubital vein, and the images of each chamber section were observed at rest and after Valsalva maneuver at least 20 cardiac cycles. Before the examination, the subjects were instructed and trained on how to perform a Valsalva maneuver by holding their breath after deep inspiration for at least 10 s, and then they were asked to forcefully exhale at the moment they heard deflation. All examined images were numbered and stored in the database; two senior sonographers selected the patients in random order for RLS source judgments and grading assessments; and finally, reliability testing of the data was performed.

c-TTE: PFO-RLS occurs the first 3–6 cycles following opacification of right atrium, and P-RLS occurs the first 3–6 cardiac cycles later (14, 15). c-TEE: PFO-RLS was considered to be present when microbubbles were observed to squeeze through the PFO, while P-RLS was considered to be present when microbubbles were observed to originate from the pulmonary veins. The grading of PFO-RLS was defined based on the following criteria: when no and when 1–10 bubbles, 11–30 bubbles, and >30 bubbles (or left atrial opacity) were detected, the P-RLS was considered to be negative, 1–30, 31–100, and >100, respectively. The P-RLS grading under c-TTE is shown in Figure 3. All subjects with P-RLS grade 3 were required to undergo computed tomographic angiography (CTA) to detect the presence of PAVMs. The images were assessed by a professional radiologist who was blinded to the study design, implementation, and data collection.

### 2.3. Statistical analysis

SPSS 25.0 statistical software (IBM Corp., Armonk, NY, United States) was used for analysis. Normally distributed data are presented as the mean ± standard deviation, and categorical data are presented as numbers (%). The chi-square test was used to compare the incidences of P-RLS detected by different methods, and the Wilcoxon rank sum test was performed for the semiquantitative grading of P-RLS. In addition, *P* < 0.05 for the difference was deemed statistically significant.

## 3. Results

### 3.1. Basic information of the study subjects

**Figure 4:**
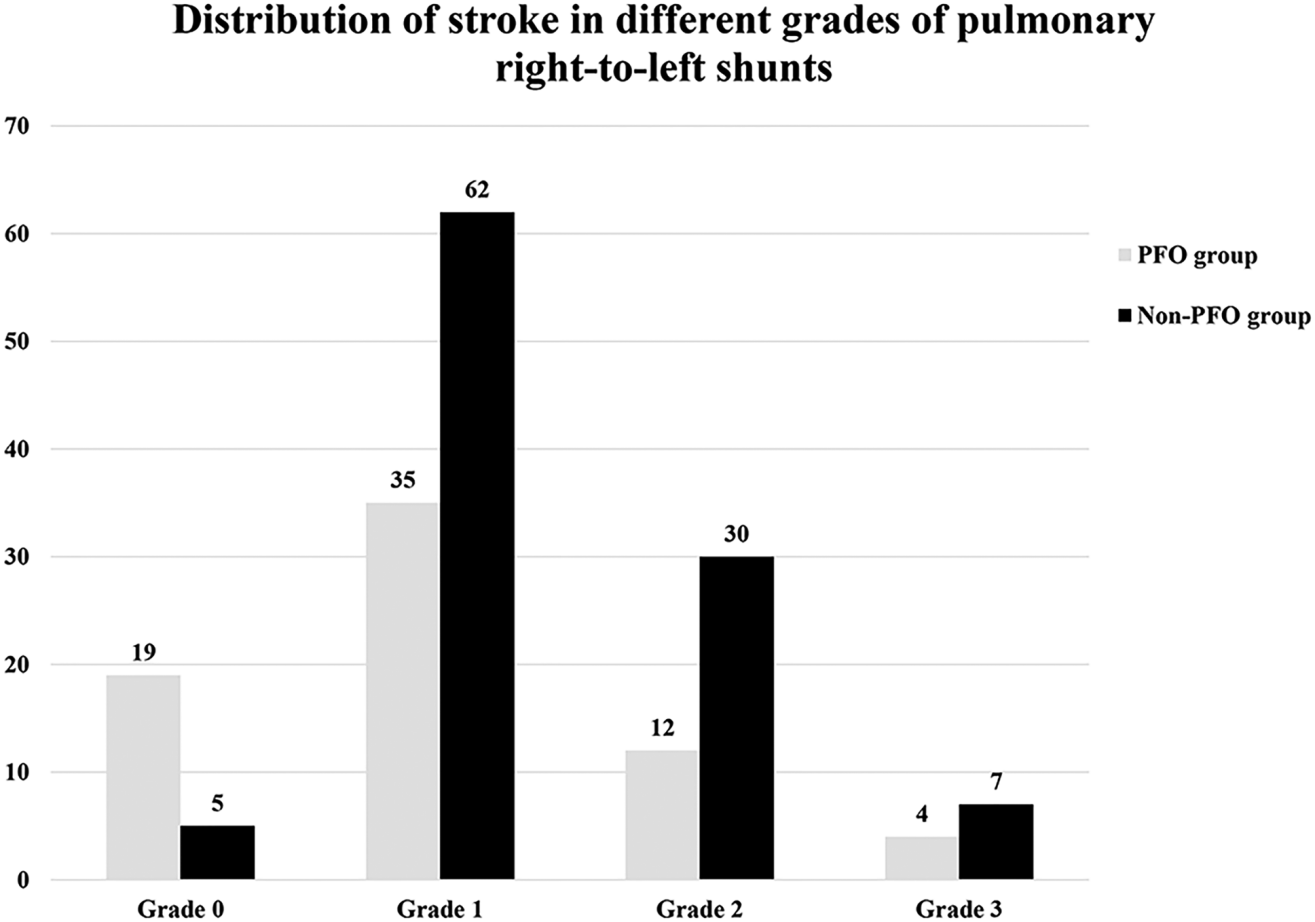
Distribution of stroke in different grades of P-RLS between PFO and non-PFO groups. P-RLS, pulmonary right-to-left shunt under c-TEE and after Valsalva maneuver; PFO, patent foramen ovale.

Overall, 407 patients were included in this study and divided into PFO group (*N* = 157, 41.0 ± 12.9 years, 47.1% male) and non-PFO group (*N* = 250, 55.7 ± 12.4 years, 56% male) according to the results of c-TEE, of which age, hypertension, diabetes, prior stroke or transient ischemic attack, migraine were different (*P* < 0.001). Interestingly, for the two groups, the incidence of stroke did not increase with P-RLS grades (16) (Table 1, Figure 4).

**Table 1:** Baseline characteristics.

Clinical characteristics	PFO group (*n* = 157)	Non-PFO group (*n* = 250)	*P*
**Age (year)**	41.0 ± 12.9	55.7 ± 12.4	0.037
**Sex (male)**	74 (47.1%)	140 (56.0%)	0.081
**Hypertension, *n* (%)**	43 (27.4%)	124 (49.6%)	0.001
**Diabetes, *n* (%)**	11 (7.0%)	65 (26.0%)	0.001
**Current smoking, *n* (%)**	42 (26.8%)	75 (30.0%)	0.481
**Coronary artery disease, *n* (%)**	2 (1.3%)	11 (4.4%)	0.081
**Prior Stroke or TIA, *n* (%)**	6 (3.8%)	55 (22.0%)	0.001
**Migraine, *n* (%)**	59 (37.6%)	32 (12.8%)	0.001
**Stroke, *n* (%)**	70 (44.6%)	104 (41.6%)	0.553
**TIA, *n* (%)**	12 (7.6%)	30 (12.0%)	0.160
**POS, *n* (%)**	3 (1.9%)	5 (2.0%)	0.950
**Dizziness/Syncope, *n* (%)**	13 (8.3%)	20 (8.0%)	0.920

Data are expressed as the mean ± SD or as the number (percentage) of patients. PFO, patent foramen ovale; TIA, transient ischemic attack; POS, platypnea-orthodeoxia syndrome.

### 3.2. P-RLS incidences under different states and examinations

In this study, the overall incidence of P-RLS was 80.3% (PFO group: 82.2%; non-PFO group: 79.2%), and the incidence of P-RLS under c-TEE was higher than that under c-TTE at rest and after Valsalva maneuver in both groups, with statistically significant results (*P* < 0.001). In the PFO group, at rest, the incidence of P-RLS under c-TEE was 73.9%, which was significantly higher than that under c-TTE (45.2%), and the difference was significant (*χ*^2^ = 26.774, *P* < 0.001). After Valsalva maneuver, the incidence of P-RLS under c-TEE was 82.2%, was significantly higher than that under c-TTE (52.2%), and the difference was significant (*χ*^2^ = 31.916, *P* < 0.001). For c-TTE and c-TEE, the incidence of P-RLS was slightly higher after Valsalva maneuver in PFO group, but the difference was not significant (c-TTE: rest vs. Valsalva maneuver, *P* = 0.214; c-TEE: rest vs. Valsalva maneuver, *P* = 0.076). In the group without PFO, the incidence of P-RLS was higher in c-TEE (at rest, 68.8%; after Valsalva maneuver 79.2%) than in c-TTE (at rest, 46.4%; after Valsalva maneuver 48.8%), both at rest and after Valsalva maneuver, and comparisons were statistically significant (*P* < 0.001). In the same modality, the Valsalva maneuver increased the incidence of P-RLS in the group without PFO, which was more significant in c-TEE (c-TTE: rest vs. Valsalva maneuver, *P* = 0.591; c-TEE: rest vs. Valsalva maneuver, *P* = 0.008) (Table 2).

**Table 2:** Comparison of the incidences of P-RLS under c-TTE and c-TEE at rest and after Valsalva maneuver [*n* (%)].

		c-TTE	c-TEE	*P*
**PFO group (*N* = 157)**	**Rest**	71 (45.2%)	116 (73.9%)	0.001
	**Valsalva**	82 (52.2%)	129 (82.2%)	0.001
	***P***	0.214	0.076	
**Non-PFO group (*N* = 250)**	**Rest**	116 (46.4%)	172 (68.8%)	0.001
	**Valsalva**	122 (48.8%)	198 (79.2%)	0.001
	***P***	0.591	0.008	

P-RLS, pulmonary right-to-left shunt; c-TTE, contrast transthoracic echocardiography; c-TEE, contrast transesophageal echocardiography; PFO, patent foramen ovale.

### 3.3. Semi-quantitative grading of P-RLS under different states and examinations

**Figure 5:**
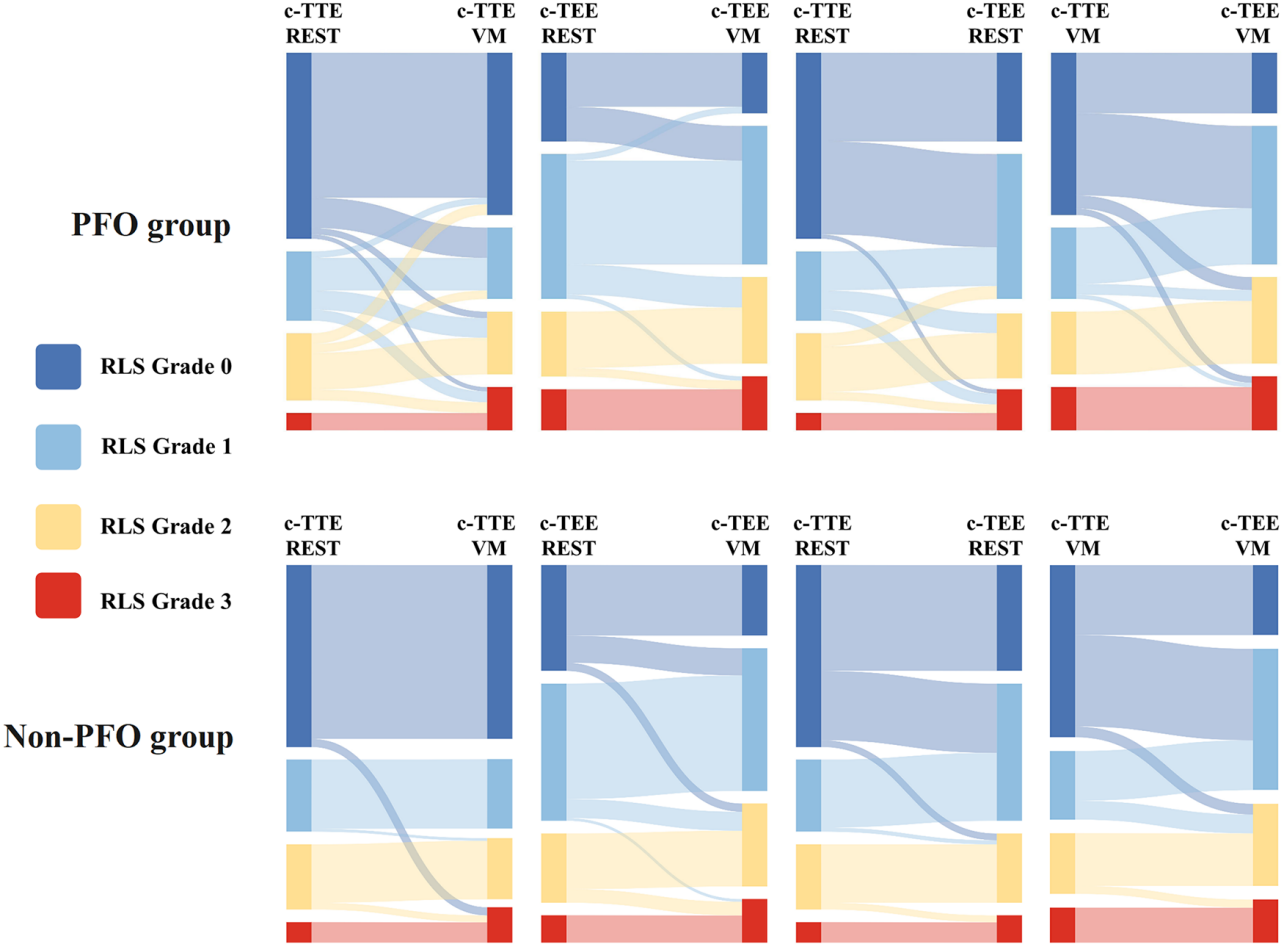
Sankey diagram of P-RLS semi-quantitative gradings between PFO and non-PFO groups. This diagram shows the trend of P-RLS grades under c-TTE and c-TEE and before and after Valsalva maneuver. RLS, right-to-left shunt; c-TTE-REST, under contrast transthoracic echocardiography and at rest; c-TTE-VM, under contrast transthoracic echocardiography and after Valsalva maneuver; c-TEE-REST, under contrast transesophageal echocardiography and at rest; c-TEE-VM, under contrast transesophageal echocardiography and after Valsalva maneuver; PFO, patent foramen ovale.

In both groups, the number of patients with high P-RLS semiquantitative grading under c-TEE was greater than that under c-TTE, and the P-RLS semiquantitative grading was significantly different between c-TTE and c-TEE in the same state (*P* < 0.001), and after c-TEE, the P-RLS grade changed most significantly from grade 0 to grade 1, while the proportion of grade 3 increased to some extent. Under the same examination method (c-TTE or c-TEE), the number of patients with high P-RLS semiquantitative grading after Valsalva maneuver was greater than that at rest, especially in c-TEE examination, the Valsalva maneuver seems to have a more significant effect on P-RLS grade changes than c-TTE. And there were significant differences in the P-RLS semiquantitative grading between the Valsalva maneuver and the resting state in both groups (*P* < 0.001) (Table 3, Figure 5).

**Table 3:** Semi-quantitative grading of P-RLS under c-TTE and c-TEE at rest and after Valsalva maneuver [*n* (%)].

		Grade 0	Grade 1	Grade 2	Grade 3
**PFO group (*N* = 157)**	**c-TTE**				
	**Rest**	86 (54.8%)	32 (20.4%)	31 (19.7%)	8 (5.1%)
	**Valsalva**	75 (47.8%)	33 (21.0%)	29 (18.5%)	20 (12.7%)
	**c-TEE**				
	**Rest**	41 (26.1%)	67 (42.7%)	30 (19.1%)	19 (12.1%)
	**Valsalva**	28 (17.8%)	64 (40.8%)	40 (25.5%)	25 (15.9%)
**Non-PFO group (*N* = 250)**	**c-TTE**				
	**Rest**	134 (53.6%)	53 (21.2%)	48 (19.2%)	15 (6.0%)
	**Valsalva**	128 (51.2%)	51 (20.4%)	45 (18.0%)	26 (10.4%)
	**c-TEE**				
	**Rest**	78 (31.2%)	101 (40.4%)	51 (20.4%)	20 (8.0%)
	**Valsalva**	52 (20.8%)	105 (42.0%)	61 (24.4%)	32 (12.8%)

P-RLS, pulmonary right-to-left shunt; c-TTE, contrast transthoracic echocardiography; c-TEE, contrast transesophageal echocardiography; PFO, patent foramen ovale.

## 4. Discussion

### 4.1. Pathophysiology of IPAVAs

IPAVAs are physiological pulmonary arteriovenous pathways with a diameter of approximately 25–50 μm (8). Currently, there is a lack of effective methods to detect IPAVAs, and can only confirm their existence through autopsy and animal experiments. It is intrinsically different from PAVMs. The origin of these inducible IPAVAs is unknown, and studies have suggested that IPAVAs may be residual nondegenerated fetal vessels that existing before birth and lose their functions after birth (17). Most of these physiological channels are distributed in the lung apex and usually not open or are less open, and when some physiological or pathological conditions occur, such as body position variation, exercise, hypoxia, etc., they will open or increase the amount of openness, resulting in P-RLS (18–20). Some studies have shown that the opening of IPAVAs is associated with migraine and stroke (21–23).

Our study found that the incidence of P-RLS was up to 82.2% in PFO patients and 79.2% in patients without PFO, and there was no statistically significant difference between the two groups. These results suggest that P-RLS has a high incidence in the population and can reach more than 80% under c-TEE and Valsalva maneuvers. This proportion is much higher than previous studies suggesting that the proportion of P-RLS that can be detected varies from 20% to 50% in healthy individuals (8, 13). For example, Lovering et al. found the proportion of P-RLS was 32% using the transplanted and fresh lungs from baboons, and Velthuis et al. found 51% P-RLS in the normal population under c-TTE. (1) Differences in detection techniques: limitations of c-TTE, which cannot wholly distinguish PFO-RLS from P-RLS, can confuse a certain proportion of P-RLS as PFO-RLS, so it improves the incidence of PFO-RLS and reduces the incidence of P-RLS. Transesophageal echocardiography, as the gold standard for PFO structural examination, combined with saline contrast echocardiography, can directly locate the PFO opening and pulmonary vein outlet and determine the source of RLS (24); (2) Our study found that the proportion of patients with P-RLS grade 1 was large, c-TTE resolution was not enough that grade 1 was easily overlooked or confused as part of PFO-RLS, while P-RLS could be clearly distinguished under c-TEE; (3) Insufficient understanding of IPAVAs, P-RLS were considered as other sources, such as PAVMs, and then RLS were included as PFO-RLS after exclusion by CTA examination.

### 4.2. Comparisons between PFO, IPAVAs and PAVMs

So far, no imaging tool can identify IPAVAs, which are detected indirectly. A grey zone probably exists between PFO, IPAVAs and PAVMs area. The incidence of PFO in patients with cryptogenic stroke is as high as 40% (3). The high prevalence of PFO in cryptogenic stroke suggested a stroke-causing role for PFO. PFO closure has been widely carried out in clinical practice as the main treatment for PFO-associated stroke. PFO-RLS often appears in association with P-RLS, and larger P-RLS is easily confused with PFO-RLS. Therefore, inaccurate assessment and neglect of IPAVAs can increase in the proportion and grade of PFO-RLS and predispose to the problem of overtreatment of PFO. Preoperative routine c-TEE is recommended (25). At the same time, we followed up with 58 patients after PFO closure in this experiment, and all patients came to our hospital for c-TTE and c-TEE within 1 month before surgery and 3 months after surgery. To assess the reliability of c-TEE in detecting P-RLS, we performed a kappa test on P-RLS before and after PFO closure in the 58 PFO patients. The kappa statistic was 0.66, indicating a substantial level of agreement. This suggests that the c-TEE on P-RLS was consistent and reliable, and increases our confidence in the validity of our findings (Table 4).

**Table 4:** Kappa test of P-RLS before and after PFO closure.

Before PFO closure	After PFO closure				
	Grade 0	Grade 1	Grade 2	Grade 3	Total
Grade 0	5	3	0	0	8
Grade 1	1	32	2	1	36
Grade 2	0	2	8	2	12
Grade 3	0	0	0	2	2
Total	6	37	10	5	58

To assess the reliability of c-TEE in detecting P-RLS, we performed a kappa test on P-RLS before and after PFO closure in 58 PFO patients. The kappa statistic was 0.66, indicating a substantial level of agreement. A kappa statistic between 0.75 and 1 indicates good agreement; a kappa statistic between 0.4 and 0.75 indicates moderate agreement; and a kappa statistic between 0 and 0.4 indicates poor agreement. P-RLS, pulmonary right-to-left shunt under c-TEE and after Valsalva maneuver; PFO, patent foramen ovale.

The pulmonary circulation pathways that generate P-RLS include PAVMs and IPAVAs. PAVMs are classified into congenital, acquired, iatrogenic, traumatic, and infectious according to their etiologies. Most of the common PAVMs are congenital pulmonary arteriovenous pathological malformations, which can be associated with hereditary hemorrhagic telangiectasia, with a very low incidence in the population; acquired PAVMs can be pathological manifestations of hepatopulmonary syndrome in the lungs; and iatrogenic PAVMs can be seen after bidirectional Green's operation for cyanotic heart disease treatment; trauma and infection lead to pulmonary arteriovenous pathway rupture, and PAVMs can also occur (26). The diameters of the PAVMs found in the clinic range from approximately 1–5 cm. They generally have a huge shunt volume that P-RLS is definitely a significant grade 3, which are easy to become the channels of ectopic thrombosis (27). Clinically larger PAVMs can be detected by CTA and are treated by minimally invasive embolization. Smaller PAVMs cannot be differentiated from IPAVAs, and no imaging tool can show the vascular anatomy, especially when the amount of P-RLS is large. However, this does not affect our study of P-RLS itself, and our study and related studies suggest that grade 1–2 P-RLS is attributed to IPAVAs present in patients, and grade 3 P-RLS may be IPAVAs or some smaller PAVMs that cannot be detected by CTA (28).

### 4.3. Associations of P-RLS with stroke

The relationship between P-RLS and stroke has been a hot spot in recent years. Some findings showed PAVMs with grade 2–3 P-RLS are related to stroke, and PAVMs with grade 1 P-RLS do not appear to lead to the occurrence of stroke (28). The relationship between IPAVAs and stroke still remains questionable. Tobin et al. and Rapp et al. have proposed IPAVAs as a possible mechanism of stroke based on necropsy and animal experiments, while some scholars suggested IPAVAs are not considered eligible for thrombus passage causing the stroke (10, 29). In our study, the incidence of P-RLS could be as high as 79.2% in patients without PFO and 41.6% of them with stroke. There was no difference in the incidence of stroke between subjects with different P-RLS grades, and our study seems to suggest that P-RLS is not associated with stroke, and changes in P-RLS grade do not lead to an increased risk of stroke (Figure 4).

The diameters of IPAVAs are approximately 25–50 μm which are functional under physiological perfusion and ventilation pressures in human, exercise-inducible IPAVAs that were between 60 and 90 μm in diameter, and studies have found that even microthrombi observed under high-resolution optical coherence tomography are no smaller than 100 μm in diameter (30, 31). Meanwhile, Gorman et al. found that when microbubbles larger than 200 μm were injected into the cerebral circulation, they would enter the vessels like long emboli rather than discretely. If these bubble emboli were longer than 5,000 μm, they were always captured but never trapped if they were <500 μm in length. Note that, in addition to size, the embolus population causing embolization must be of a certain length (16). At the same time, it has been found that the human body has a dynamic cerebral autoregulation that can filter some subclinical or emboli and protect the body to some extent (29). The above evidence supports that IPAVAs cannot lead to stroke.

### 4.4. Influences of c-TEE on P-RLS compared with c-TTE

In our study, two phenomena were noticed (Table 2): (1) regardless of whether the participants were at rest or after Valsalva maneuver, the incidence and shunt volume of P-RLS observed by c-TEE in the subjects with P-RLS were increased to different extents compared with c-TTE, both statistically different (*P* < 0.001). The proportion of P-RLS at each level was rearranged, in particular, the proportion of grade 1 P-RLS increased most significantly, and the number of overall P-RLS high grades of c-TEE was increased compared with c-TTE (Figure 5, Table 3), except the reasons that c-TEE itself has a higher resolution for RLS than c-TTE and can directly distinguish its source (32). Moreover, also considering that it may be during c-TEE, the stimulating effects of the transesophageal echocardiography probe on IPAVAs, patient anxiety or pharyngeal hypersensitivity lead to hypoxia (20). It was reported in the literature that respiratory secretions and tongue base retropulsion are also the main causes of hypoxia during c-TEE, especially in obese patients, who are most likely to have tongue retropulsion, respiratory obstruction, plus transesophageal echocardiography probe stimulations to induce laryngospasm, which can finally lead to hypoxia (33). (2) The incidence and shunt volume of P-RLS also increased to some extent after Valsalva maneuver in some subjects compared with the resting state. The mechanism differs from the Valsalva maneuver, which increases PFO-RLS shunt volume by increasing the return blood volume (34). The mechanism is currently unknown, possibly due to multiple and prolonged strenuous suction, suffocation, physical exertion, patient tension, or even a hypoxic state during the training and requiring the participants to repeat the Valsalva maneuver that induces the opening or increasing the opening of the IPAVAs leading to an increase in the incidence and shunt volume of P-RLS.

### 4.5. Comparisons between two P-RLS quantitative classifications

**Figure 6:**
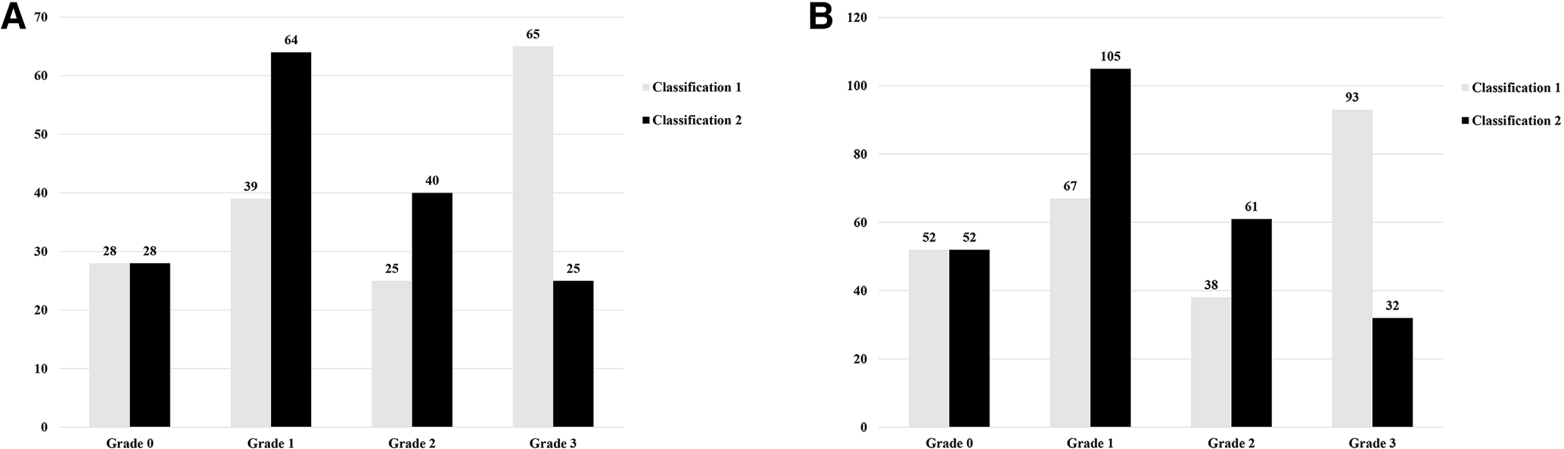
Comparison of two P-RLS quantitative classifications in PFO group (**A**); comparison of two P-RLS quantitative classifications in non-PFO group (**B**). P-RLS, pulmonary right-to-left shunt under c-TEE and after Valsalva maneuver; PFO, patent foramen ovale. Classification 1: grade 0 = no bubbles, grade 1 = 1–10 bubbles, grade 2 = 11–30 bubbles, and grade 3 > 30 bubbles (or left atrial opacity) were detected; Classification 2: grade 0 = no bubbles, grade 1 = 1–30 bubbles, grade 2 = 31–100 bubbles, and grade 3 > 100 bubbles (or left atrial opacity) were detected, respectively.

There are currently two P-RLS quantitative classifications: Classification 1: the P-RLS grade is the same as the PFO-RLS grade, and Classification 2: the P-RLS grade is relatively lower than the PFO-RLS grade (35). In this study, Classification 2 was mainly used, and the two quantitative classifications were demonstrated by c-TEE during the research process. Our study found that when P-RLS was analyzed by Classification 1, 41.4% (65/157) of the subjects with P-RLS grade 3 after Valsalva maneuver in the PFO group and 37.2% (93/250) in the non-PFO group, respectively, and the proportion was high. However, when P-RLS was analyzed by Classification 2, only 15.9% (25/157) and 12.8% (32/250) of the subjects with P-RLS grade 3 after Valsalva maneuver in the PFO group and non-PFO group, respectively, and the remaining subjects were P-RLS grade 1 or 2 (Table 5, Figure 6). After the CTA examination, only four patients with PAMVs was found in the two groups. Therefore, this study concluded that Classification 2 is more reasonable than Classification 1 and plays a role in screening PAVMs to a certain extent. By using this technique, fewer patients receive CTA examinations, avoiding unnecessary exposure to ionizing radiation and using iodinated contrast agents. At the same time, we consider PFO-RLS and P-RLS as two shunts of different properties and deserve different grading criteria.

**Table 5:** Comparison of two P-RLS quantitative classifications between PFO and non-PFO groups.

PFO group			Non-PFO group		
	Classification 1	Classification 2		Classification 1	Classification 2
**Grade 0**	28	28	**Grade 0**	52	52
**Grade 1**	39	64	**Grade 1**	67	105
**Grade 2**	25	40	**Grade 2**	38	61
**Grade 3**	65	25	**Grade 3**	93	32

Classification 1: grade 0 = no bubbles, grade 1 = 1–10 bubbles, grade 2 = 11–30 bubbles, and grade 3 > 30 bubbles (or left atrial opacity) were detected; Classification 2: grade 0 = no bubbles, grade 1 = 1–30 bubbles, grade 2 = 31–100 bubbles, and grade 3 > 100 bubbles (or left atrial opacity) were detected, respectively. P-RLS, pulmonary right-to-left shunt under c-TEE and after Valsalva maneuver; PFO, patent foramen ovale.

## 5. Limitations

The results of this study have great clinical implications, but some limitations still need to be considered. (1) Our study is a single-center study, and the sample size included is limited. The study should be continued with a larger sample size and a combined multicenter design. (2) Our study design conditions were limited, and other P-RLS related influencing factors, such as body position, exercise, and hypoxia, were not investigated. (3) Due to the lack of effective IPAVAs detection methods, only reflect the P-RLS rules through the relevant indirect signs, so larger IPAVAs cannot be distinguished from PAVMs which cannot be detected by CTA when shunt volume is grade 3.

## 6. Conclusion

Our study showed that the vast majority of P-RLS are grade 1–2 and are derived from physiological IPAVAs, which do not affect the management disposal decisions for PFO and should be considered clinically insignificant IPAVAs. However, the incidence of P-RLS is higher than that of PFO-RLS in the normal population, and the combined appearance of PFO-RLS and P-RLS is very common; therefore, attention should be given to the differentiation between P-RLS and PFO-RLS. c-TEE is an effective method to detect P-RLS. However, P-RLS can be stimulated under TEE and after Valsalva maneuver, so this effect should be noted.

## Data Availability

The raw data supporting the conclusions of this article will be made available by the authors, without undue reservation.
